# Imaging Manifestations of Intrahepatic Reactive Lymphoid Hyperplasia: A Case Report and Literature Review

**DOI:** 10.3389/fonc.2021.694934

**Published:** 2021-12-09

**Authors:** Bingqian Zhang, Mingyue Zou, Zengxin Lu, Haijia Mao, Ya’nan Huang, Fang Liu, Zhenhua Zhao

**Affiliations:** ^1^ Department of Radiology, Shaoxing People’s Hospital (Shaoxing Hospital, Zhejiang University School of Medicine), Shaoxing, China; ^2^ Department of Pathology, Shaoxing People’s Hospital (Shaoxing Hospital, Zhejiang University School of Medicine), Shaoxing, China

**Keywords:** reactive lymphoid hyperplasia, hepatocellular tumor, pseudo-lymphoma, case report, hepatectomy

## Abstract

Reactive lymphoid hyperplasia (RLH) of the liver is a rare benign disease. This article describes a 77-year-old female patient with RLH of the liver. The patient was admitted to the hospital due to atrial fibrillation. A liver tumor was incidentally found during abdominal enhanced CT. Further magnetic resonance imaging (MRI) and PET/CT showed four lesions in the liver. The imaging findings suggested hepatocellular carcinoma (HCC), but it was not consistent that the patient had no history of liver cirrhosis and hepatitis, and a variety of tumor markers were within the normal range. The largest lesion was surgically removed and microscopically diagnosed as RLH of the liver. The pathology included a large number of reactive hyperplastic lymphoid follicles. Immunohistochemical examination showed that the infiltrating lymphocytes were polyclonal. The authors believe that the perinodular enhancement on MRI, the obvious limitation of diffusion on DWI, the insignificant increase of SUVmax on PET-CT delayed phase, and the support of clinical data can help distinguish liver RLH from lymphoma and HCC.

## Introduction

Reactive lymphoid hyperplasia (RLH) is first reported in 1981 by Snover et al., also known as pseudo-lymphoma or nodular lymphoid lesion (1). RLH has been reported in various organs including the skin, lung, gastrointestinal tract, intraventricle, and rarely in the liver (2–5). RLH of the liver is characterized on histopathology by the proliferation of non-neoplastic, polyclonal lymphocytes forming follicles with an active germinal center (6). The etiology is still unknown, and the pathogenesis remains unclear, but it is speculated that RHL may be related to the reactive immunological response to inflammatory process or chronic infection and may be associated with malignant tumor (7, 8). Due to the imaging manifestations and laboratory results of RLH are not specific, it is difficult to differentiate RLH from liver metastases and hepatocellular carcinoma (HCC) in imaging, and it is also difficult to differentiate RLH from lymphoma in pathology (9). The diagnosis usually had not been made until a pathological examination was performed after surgical resection. Here, we report a case of intrahepatic RLH with no obvious clinical symptoms and discuss the clinical and histological features, etiology, and imaging findings in conjunction with the literature review.

## Case Presentation

**Figure 1:**
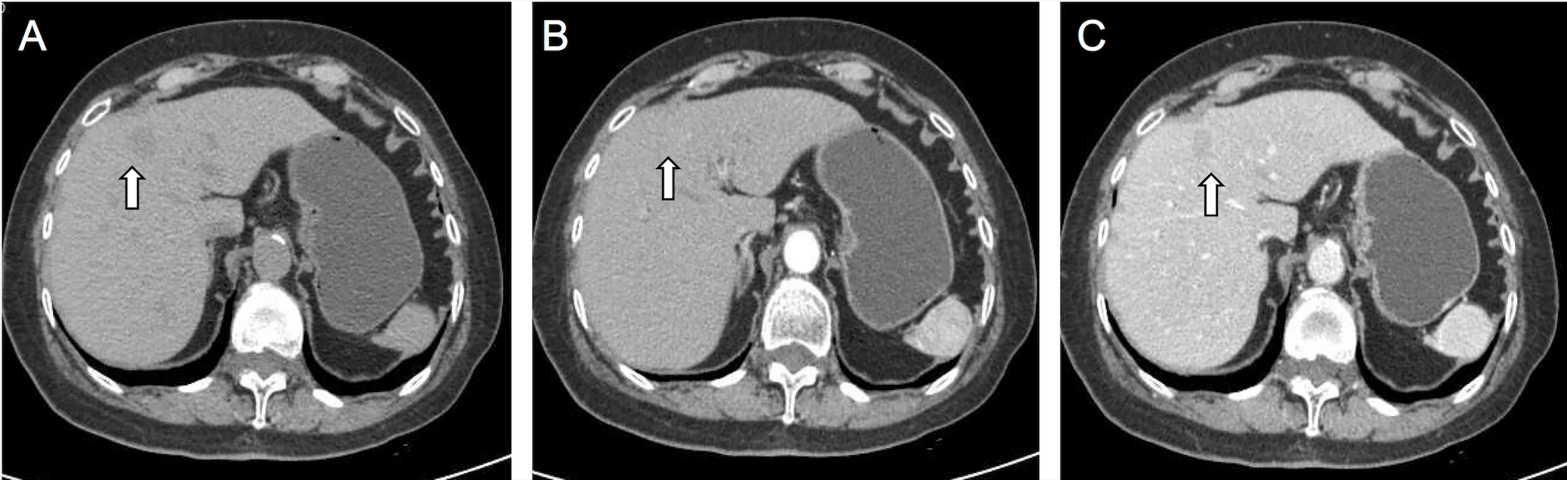
On CT plain image, the nodule is slightly low-density lesion in the S4 of the liver **(A)**. On contrast-enhanced CT images, the lesion showed slight enhancement in arterial phase **(B)** and slight washout in portal phases **(C)**. The white arrow points to the lesion. Malignant tumor could not be excluded.

A 77-year-old woman was hospitalized in the cardiology department due to atrial fibrillation. Contraindications were excluded, and atrial fibrillation (AF) ablation was performed on an optional schedule. However, abdominal contrast-enhanced computed tomography (CT) scan found a circular and low-density lesion in the Segment 4 of the liver with unclear border, approximately 15 mm× 12 mm in size, which was mild progressive enhanced in the arterial phase and portal phase. HCC was suspected, but no hepatic cirrhosis and history of hepatitis (**Figure 1**). Tumor markers, including CEA, CA199, CA125, AFP, were within normal range, hepatitis panel was negative, and hepatic function was normal.

**Figure 2:**
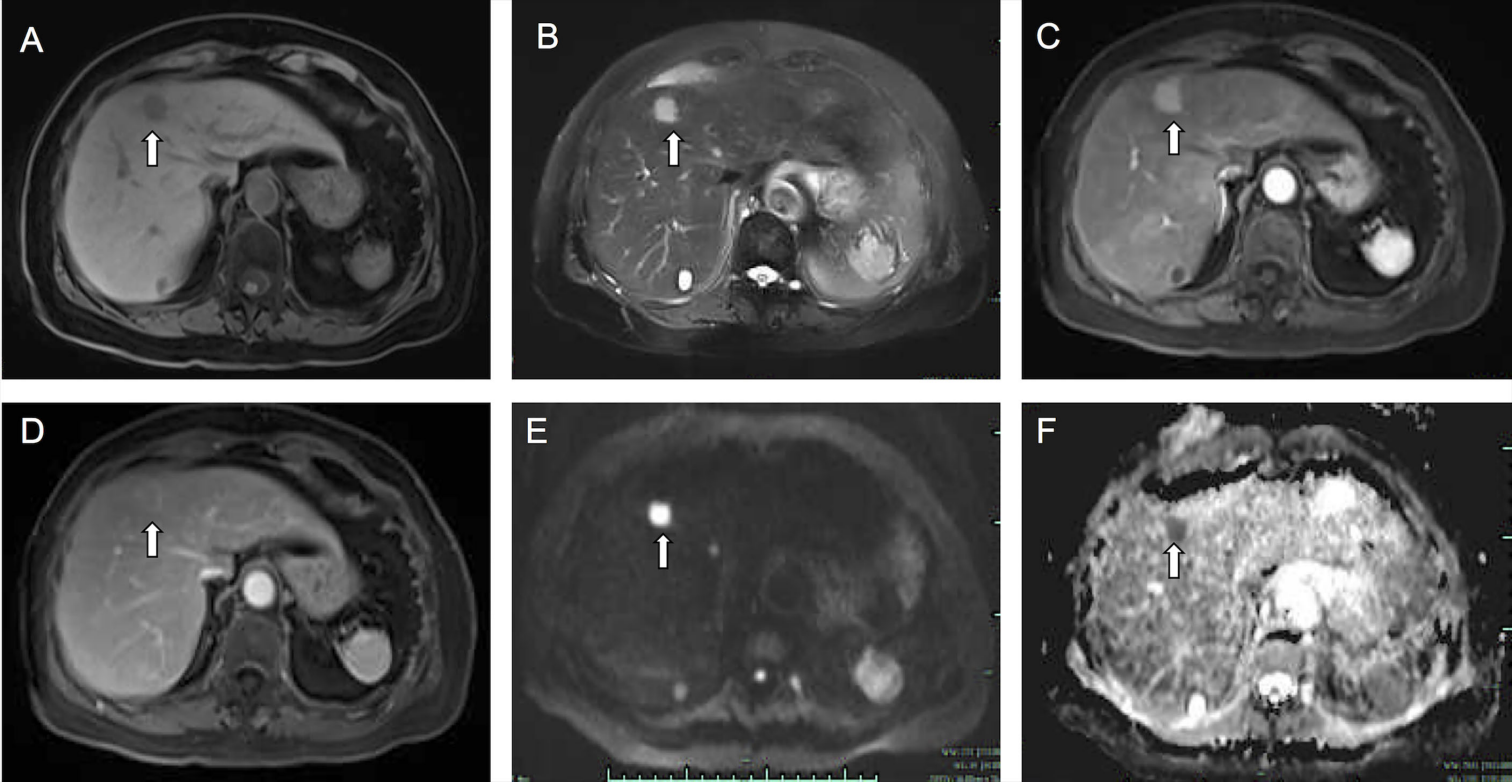
Fat-saturated T1-weighted image **(A)** showed a hypointense lesion measuring 17 mm in the segment 4 of the liver. The lesion showed hyperintensity on T2-weighted image **(B)**. On enhanced T1-weighted images, the lesion showed obvious enhancement in arterial phase **(C)**, and in portal phase **(D)** the lesion showed slight washout and perinodular enhancement. On DWI **(E)** and ADC **(F)** images, the lesion showed a significant diffusion restriction. The white arrow points to the lesion.

To confirm the diagnosis, the patient underwent further examinations. Abdominal magnetic resonance imaging (MRI) disclosed one nodule in the segment 7, one nodule in the segment 5, and two nodules in the segment 4 of the liver (4, 6, 17, and 6 mm in diameter), respectively. The larger one was located in the segment 4 of liver, with hypointense on T1-weighted images (T1WI) and hyperintense on T2-weighted images (T2WI), which showed significant enhancement on the arterial phase and slight washout on the portal phase. In the delayed phase, the edge of the tumor was underscored as a circular enhancement which is unsimilar to the enhancement method of dynamic CT. It showed a significant restriction of diffusion on diffusion weighted images (DWI) and apparent diffusion coefficient (ADC). Other lesions in segments 7 and 5 showed the same hemodynamic characteristics as the tumor in segment 4 (**Figure 2**). Based on MRI findings, it is considered as tumorous lesions of the liver.

**Figure 3:**
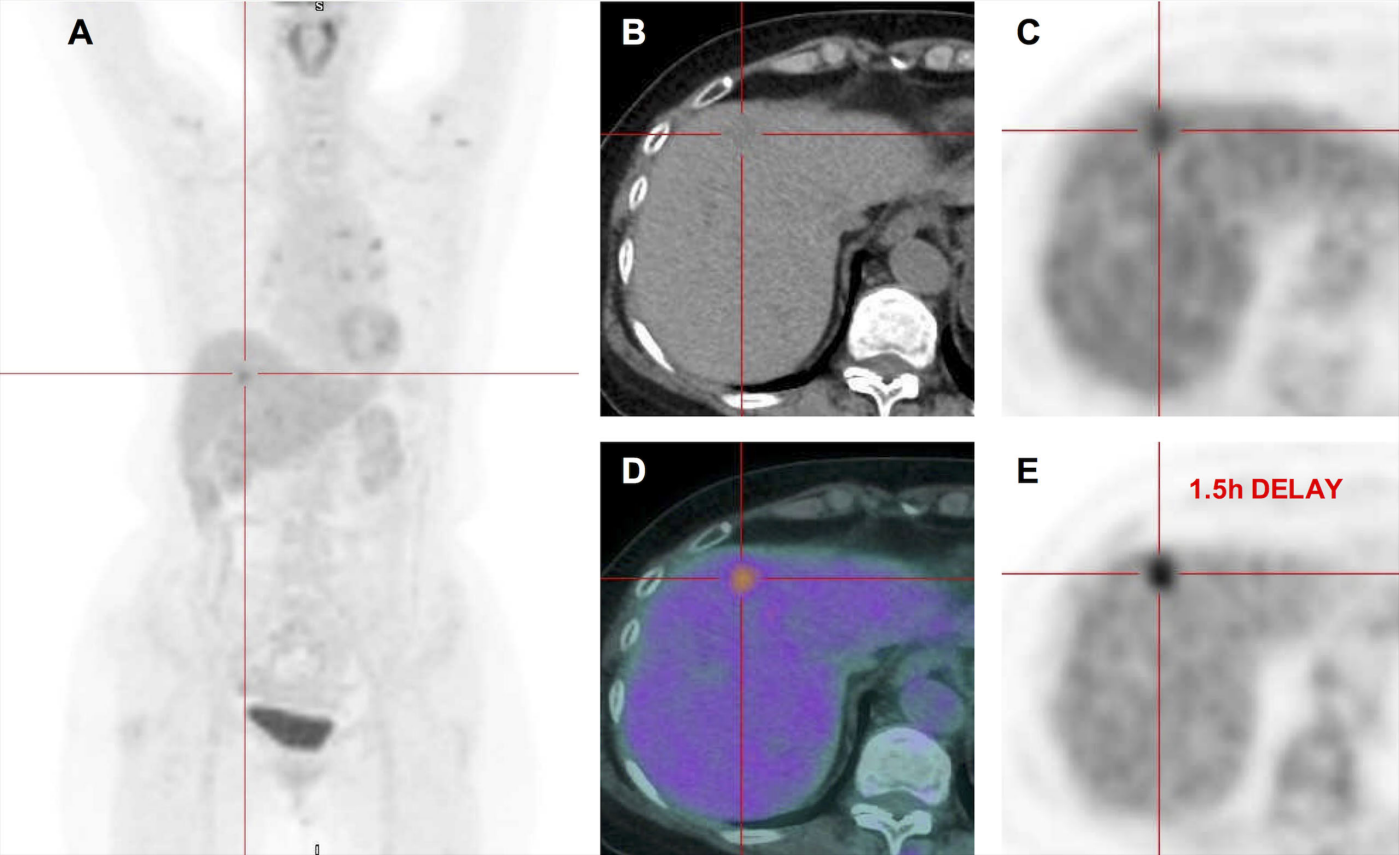
^18^FDG PET-CT images. Maximum intensity projection PET **(A)**. Transverse CT **(B)** showed a slightly low-density lesion in segment 4 of liver. Corresponding PET **(C)** and fused **(D)** images showed increased FDG uptake with SUVmax of 4.81. Corresponding PET image **(E)** after 1.5 h delay showed the FDG uptake that continued to increase with SUVmax of 5.24, and the detention index was 8.9%. However, the lesions in the S7 and S5 of the liver showed no significant increase in FDG uptake.

For further differential diagnosis, the patient underwent positron emission tomography-computer tomography (PET-CT) examination, founding that the segment 4 of liver had a slightly low-density lesion with increased uptake of 18F-fluoro-deoxy-glucose (FDG), and SUVmax of early and delayed enhancement imaging was 4.81 and 5.24, respectively. The detention index was 8.9%. However, the lesions in segments 7 and 5 of the liver showed no significant increase in FDG uptake (**Figure 3**). As all images suggested HCC, the medial segment of the liver was finally surgically removed.

**Figure 4:**
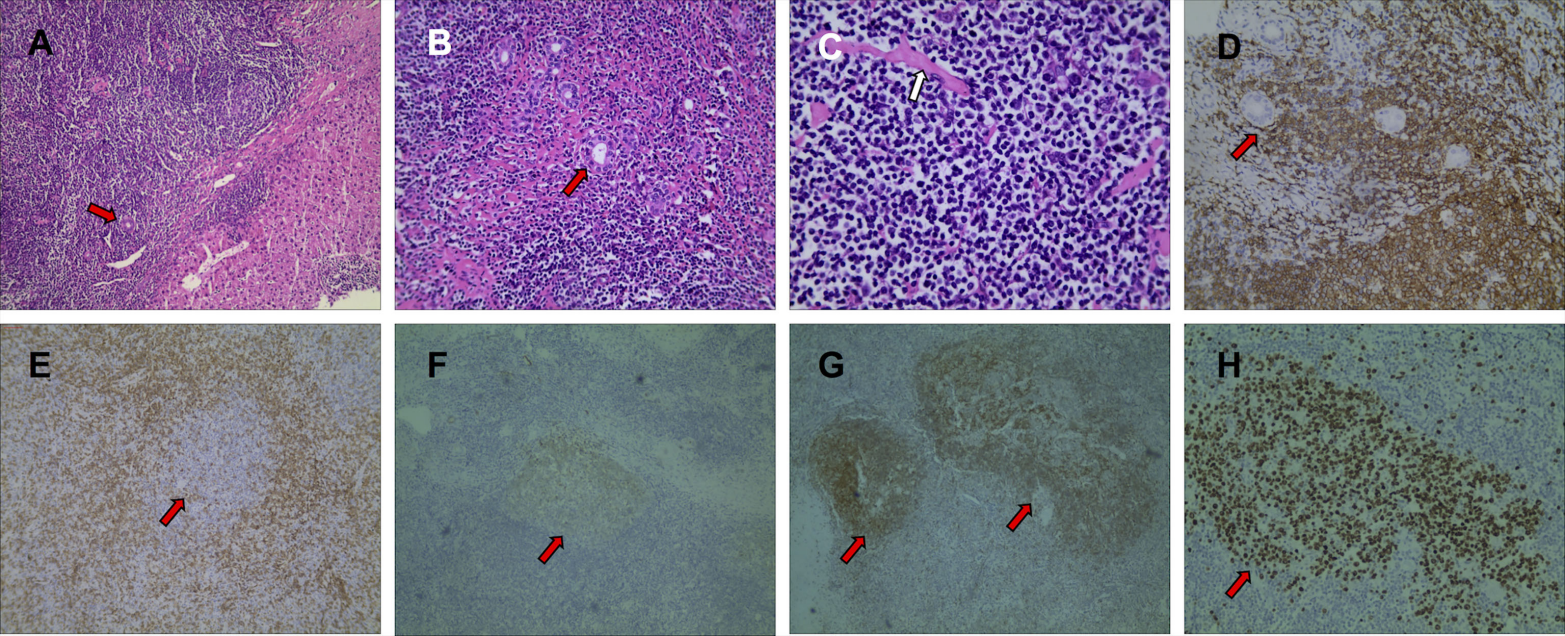
Pathological findings with hematoxylin-eosin (HE) staining showed that there were a large number of reactive proliferative lymphoid follicles in the tumor area, and the germinal center was composed of non-dysmorphic lymphocytes or plasma cells, fibrous collagen, and hyaluronic matrix, and remaining normal bile ducts (red arrows) can be seen along the edge of the lesion (**A**, HE×100). Lymphoid tissues grow around the small bile ducts (**B**, HE×200). A uniform red-stained matrix deposit can be seen in the lesion (**C**, HE×400). The results of immunohistochemistry showed that CD20-positive lymphocytes grew around the small bile ducts (**D**, IHC×200). CD3 (**E**, IHC×100), CD10 (**F**, IHC×40), CD23 (**G**, IHC×40), Ki-67 (**H**, IHC×40) staining showed reactive proliferative lymphoid follicles in the lesion.

Surgical removal of a piece of left medial lobe of liver tissue, about 6 cm × 4 cm × 2.5 cm in size, the cut surface shows an expanded lumen structure, and a gray-white mass in the liver parenchyma. The size is about 2.5 × 2cm, part of the boundary is unclear, and the texture is soft. The HE-stained sections under low-power microscope showed that massive lymphoid tissues proliferated in the lesion and formed massive lymphoid follicles, and the peripheral lymphoid tissues of the lesion grew around the small bile ducts, but no clear lymphoepithelial lesions were seen. The interfollicular area is dominated by small lymphocytes, and many plasma cells can be seen. In the lymphatic tissue, there were homogeneous red-stained collagen-like deposits around some small blood vessels. Immunohistochemical results were as follows: Bcl-2(−), Bcl-6(germinal center +), CD10(germinal center +), CD20(+), CD23(+), CD3(+), Ki-67(germinal center +), CD5(+), CyclinD1(−), TDT(−), kappa/lambda ratio about 2:1. EBV negative by qRT-PCR method (**Figure 4**). A genetic study of the immunoglobulin heavy chain (IgH) clonality on the DNA of tumor tissues was performed using capillary electrophoresis method, which was reported previously (10). The results showed that no clonal IgH gene rearrangement was detected: IGH (−), IGK (−), IGL (−). Therefore, the possibility of extranodal marginal-zone lymphoma (MALT) was ruled out, and the final diagnosis was RLH. Combined with imaging, pathology, and clinical manifestations, the clinical diagnosis was multifocal RLH in the liver. Three months after the operation, a re-examination of the abdominal ultrasound showed no signs of recurrence.

## Discussion

Intrahepatic RLH is a rare benign non-specific disease. Its pathological characteristics are the formation of massive lymphoid follicles and active germinal centers, and there is no obvious atypia of lymphocytes. Intrahepatic RLH is a rare benign lesion, which is pathologically characterized by hyperplastic lymphoid follicles with reactive germinal centers (11). Although the pathogenesis of RHL remains unknown, many cases have been reported to be associated with chronic inflammation, autoimmune diseases, and malignant tumors (12–14). A previous case reported that intrahepatic RLH developed after interferon α treatment for chronic hepatitis B, suggesting that it is related to chronic inflammation and immunosuppressive agents (13). However, this patient did not have any autoimmune diseases or malignant tumors.

The imaging findings of the intrahepatic RLH were non-specific. On enhanced CT, it showed mild progressive enhancement. On enhanced MRI, it showed significant enhancement on the arterial phase, slight washout on the portal phase, and perinodular enhancement on the delayed phase. The authors believe that perinodular enhancement on the delayed phase may be one of the characteristic manifestations of RHL, because HCC often manifests as contrast agent washout in the delayed phase without perinodular enhancement. Even so, it is still difficult to rule out the possibility of highly differentiated malignant tumors. On PET/CT, the SVUmax of intrahepatic RLH was increased, which behaved similarly to intrahepatic HCC and liver MALT lymphoma (15, 16). Therefore, it is very difficult to differentiate intrahepatic RLH from HCC, metastatic tumor, liver lymphoma, and other malignant tumors only by imaging findings. Ultimately, surgical resection or preoperative biopsy was therefore an essential diagnostic tool.

Machida T et al. considered that the “perinodular enhancement” of lesions on enhanced CT was a prominent feature of RHL, but whether this feature has diagnostic value needs further study (6). Periodontal enhancement was not seen in the enhanced CT image of the present case, but this feature was seen in enhanced MRI. In order to differentiate from liver malignant tumors, attention should be paid to the clinical characteristics of the patient. In this case, it is especially worth noting that the patient has no background of cirrhosis and hepatitis, and AFP was negative, and the intrahepatic lesions were discovered accidentally. In addition, according to a statistic, RLH mainly occurs in middle-aged women, with an average age of 54.1 years (6). Thus, when a female patient accidentally finds liver lesion without a background of liver cirrhosis or chronic viral hepatitis infection, especially when tumor markers such as AFP are normal, intrahepatic RLH must be indicated in the differential diagnosis (17, 18).

Considering that RLH is a rare disease, the PET/CT performance of RLH reported in the literature was even rarer. It has been previously described that FDG uptake in the localized reactive lymphoid hyperplasia of the spleen, with a SUVmax of 4.3 (19). Zhong X et al. reported the PET/CT manifestations of two cases of intrahepatic RLH, which displayed increased FDG uptake, and the SUVmax were 6.6 and 6.7, respectively (20). PET/CT can help distinguish malignant and benign liver lesions, but there is a possibility of false positives. Although it is rare, hepatic reactive lymphoproliferation is one of the causes of false-positive results. In our case, the SUVmax of the largest liver lesion on PET/CT was 4.81, which mimicked malignancy.

It is difficult to accurately identify liver RLH only through routine histological evaluation, and it must be distinguished from malignant lymphoma. Immunohistochemistry and molecular genetics research are necessary to distinguish them. In this case, immunohistochemical examination and IG gene clonal rearrangement examination were performed to rule out lymphoma. The active proliferation of small B lymphoid cells, the proliferation of lymphoid follicles, including the differentiation of plasma cells, suggest that extranodal marginal zone lymphoma cannot be ruled out. Kappa/lambda ratio about 2:1, B cell markers (CD20+) and T cell markers (CD3+) indicate polyclonality. In addition, based on the negative results of bcl-2, the reproductive center is not considered a tumor. The results of genetic investigations on the clonal rearrangement of the IG gene also support histological findings. Therefore, the diagnosis of liver RLH seems reasonable.

## Conclusion

In conclusion, intrahepatic RLH should be considered in the differential diagnosis of small liver tumors, especially in female patients and patients without risk factors for liver cancer. The author believes that the perinodular enhancement on MRI, the obvious limitation of diffusion on DWI, the insignificant increase of SUVmax on PET-CT delayed phase, and the support of clinical data can help distinguish liver RLH from lymphoma and HCC before surgery, but it does exist many cases cannot be clearly identified before surgery. Therefore, it is very important to follow up the patients closely, and surgical treatment should be selected for suspicious malignant tumors. 

## Data Availability Statement

The original contributions presented in the study are included in the article/supplementary material, further inquiries can be directed to the corresponding author.

## Ethics Statement

The studies involving human participants were reviewed and approved by the Institutional Review Board of Shaoxing People’s Hospital. The patients/participants provided their written informed consent to participate in this study. Written informed consent was obtained from the individual(s) for the publication of any potentially identifiable images or data included in this article.

## Author Contributions

BZ and MZ composed the manuscript and literature review. FL provided figures and pathology review. ZL, HM, and YH had the acquisition, analysis, or interpretation of data for the work. ZZ revised it critically for important intellectual content, gave final approval of the version to be published, and agreed to be accountable for all aspects of the work in ensuring that questions related to the accuracy or integrity of any part of the work are appropriately investigated and resolved.

## Funding

This work was supported by Public Welfare Technology Research Project in Zhejiang Province of China (Grant No. LGF19H220002) and Key Laboratory of Functional Molecular Imaging of Tumor and Interventional Diagnosis and Treatment of Shaoxing City. All funding departments had no role in the collection, analysis, or interpretation of the data or in the decision to submit the manuscript for publication.

## Conflict of Interest

The authors declare that the research was conducted in the absence of any commercial or financial relationships that could be construed as a potential conflict of interest.

## Publisher’s Note

All claims expressed in this article are solely those of the authors and do not necessarily represent those of their affiliated organizations, or those of the publisher, the editors and the reviewers. Any product that may be evaluated in this article, or claim that may be made by its manufacturer, is not guaranteed or endorsed by the publisher.
